# Elective cesarean section for women living with HIV: a systematic review of risks and benefits

**DOI:** 10.1097/QAD.0000000000001535

**Published:** 2017-06-28

**Authors:** Caitlin E. Kennedy, Ping T. Yeh, Shristi Pandey, Ana P. Betran, Manjulaa Narasimhan

**Affiliations:** a Department of International Health, Johns Hopkins Bloomberg School of Public Health, Baltimore, Maryland, USA; b Department of Reproductive Health and Research, World Health Organization, including the UNDP, UNFPA, UNICEF, WHO, World Bank, Special Programme of Research, Development and Research Training in Human Reproduction (HRP), Geneva, Switzerland.

**Keywords:** cesarean-section, elective cesarean-section, maternal health, mode of delivery, mother-to-child transmission, reproductive health, vertical transmission

## Abstract

**Objective and design:**

To inform WHO guidelines, we conducted a systematic review and meta-analysis to assess maternal and perinatal outcomes comparing cesarean section (c-section) before labor and rupture of membranes [elective c-section (ECS)] with other modes of delivery for women living with HIV.

**Methods:**

We searched PubMed, CINAHL, Embase, CENTRAL, and previous reviews to identify published trials and observational studies through October 2015. Results were synthesized using random-effects meta-analysis, stratifying for combination antiretroviral therapy (cART), CD4^+^/viral load (VL), delivery at term, and low-income/middle-income countries.

**Results:**

From 2567 citations identified, 36 articles met inclusion criteria. The single randomized trial, published in 1999, reported minimal maternal morbidity and significantly fewer infant HIV infections with ECS [odds ratio (OR) 0.2, 95% confidence interval (CI) 0.0–0.5]. Across observational studies, ECS was associated with increased maternal morbidity compared with vaginal delivery (OR 3.12, 95% CI 2.21–4.41). ECS was also associated with decreased infant HIV infection overall (OR 0.43, 95% CI 0.30–0.63) and in low-income/middle-income countries (OR 0.27, 95% CI 0.16–0.45), but not among women on cART (OR 0.82, 95% CI 0.47–1.43) or with CD4^+^ cell count more than 200/VL less than 400/term delivery (OR 0.59, 95% CI 0.21–1.63). Infant morbidity moderately increased with ECS.

**Conclusion:**

Although ECS may reduce infant HIV infection, this effect was not statistically significant in the context of cART and viral suppression. As ECS poses other risks, routine ECS for all women living with HIV may not be appropriate. Risks and benefits will differ across settings, depending on underlying risks of ECS complications and vertical transmission during delivery. Understanding individual client risks and benefits and respecting women's autonomy remain important.

## Abstract 2

Supplemental Digital Content is available in the text

## Introduction

Cesarean section (c-section) before labor and before rupture of membranes [elective c-section (ECS)] has been suggested as an intervention to prevent vertical transmission of HIV in high-income settings where training and resources exist to conduct c-sections safely [1]. The decision to offer ECS to women living with HIV must consider a range of potential risks as well as benefits for both the mother and the child. These risks and benefits vary depending on the underlying risk of vertical transmission of HIV during delivery, which is associated with disease stage and antiretroviral treatment (ART) use [2], as well as on the underlying risks of ECS compared with vaginal delivery for both mother and child, which is associated with the local capacity and skills to perform c-sections and treat potential complications [3]. Unfortunately, many women in low-income and middle-income countries (LMICs), in particular, lack access to high-quality obstetric services, a critical concern in the context of rising c-section rates globally [3]. Furthermore, women living with HIV may experience higher rates of some obstetric complications compared with HIV-uninfected women [4].

In 2005, Read and Newell published a Cochrane systematic review, which identified one clinical trial and five observational studies evaluating the safety of ECS versus vaginal delivery among HIV-1-infected women [5]. Taken together, these studies indicated that ECS can substantially reduce the risk of mother to child HIV transmission, whereas it also resulted in slightly higher rates of postpartum maternal morbidity, such morbidity was generally rated as minor [5]. The authors concluded that in general, the benefit of ECS outweighs the risks, but the risk-to-benefit ratio depends upon the underlying rate of vertical HIV transmission [5].

There were several limitations to the data available at the time of the Read and Newell review [5] as well as to its interpretation and applicability 12 years later. The single trial included only HIV-1-infected women taking no ART during pregnancy or taking only zidovudine. In addition to this, HIV infection, no infant outcomes were measured in any of the included studies. Furthermore, all studies were conducted in high-income countries in Europe or North America, where ECS is a relatively safe procedure. The vast majority of women living with HIV live in sub-Saharan Africa and other LMIC settings, where higher rates of morbidity and mortality may be ascribed to the c-section surgery itself. Since the single trial, published in 1999 with data collected in the mid-1990s, ART use has expanded greatly worldwide, and more effective regimens have been developed. In 2015, the WHO recommended offering immediate ART to all individuals living with HIV [2]. These actions should significantly reduce vertical HIV transmission.

Women living with HIV have the right to the most up-to-date knowledge about risks and benefits of sexual and reproductive health decisions they will make, with the support of their healthcare providers [6]. To inform WHO recommendations on the sexual and reproductive health and rights of women living with HIV, we sought to update the Read and Newell review [5] to consider the current existing evidence on ECS for women living with HIV globally.

## Methods

This systematic review and meta-analysis followed Preferred Reporting Items for Systematic Reviews and Meta-Analyses (PRISMA) guidelines [7,8] to answer the question: does ECS in women living with HIV result in better maternal and perinatal outcomes than other modes of delivery?

### Inclusion/exclusion criteria

To be included in the review, an article had to present primary research comparing outcomes of ECS to other modes of delivery (e.g. non-ECS, vaginal delivery) among women living with HIV and their children; measure any of the following outcomes: morbidity and mortality among women [e.g. febrile morbidity, endometritis, hemorrhage or severe anemia, pneumonia, urinary tract infections (UTIs)], HIV infection in infants (efficacy of prevention of vertical transmission), other morbidity and mortality among infants (e.g., respiratory morbidity and skin lacerations), or breastfeeding (success or timing of initiation and continuation); and be published in a peer-reviewed journal prior to the search date of 1 October 2015. Analytic epidemiologic studies, both observational (case–control and cohort studies) and interventional (clinical trials), were included; ecological and historical-control studies were not. Mode of delivery had to be explicitly described. Studies from any geographical location including any women living with HIV of childbearing age were eligible for inclusion. Studies published in all languages were eligible for inclusion.

For this review, we defined ECS as a c-section conducted before start of labor and before rupture of membranes. However, we included any study that used the term ECS, without requiring further definition by study authors. We similarly accepted author-provided definitions for all outcomes.

### Search strategy

We searched four electronic databases: PubMed, CINAHL, Embase, and the Cochrane Central Register of Controlled Trials (CENTRAL). For each online database, we used the following search strategy: (HIV OR AIDS) AND (‘mode of delivery’ or ‘cesarean section’ or ‘cesarean section’ or ‘c-section’). We conducted secondary reference searching on all included studies and the previous Read and Newell review [5].

Titles, abstracts, citation information, and descriptor terms of citations identified through the search strategy were screened by a member of the study staff. When a citation was considered relevant or when title/abstract was deemed insufficient for inclusion/exclusion decision, the full-texts were retrieved and evaluated. Two reviewers (independently and in duplicate) assessed all full-text articles for eligibility to determine final study selection. Differences were resolved through consensus.

### Data extraction and quality assessment

Data were extracted by two reviewers using standardized forms. Differences in data extraction were resolved through discussion and referral to a senior study team member when necessary. The following information was gathered from each included study:

(1) Study description: Study objectives; year(s); location (country/city); setting (population-based, hospital, clinic); study design; sample size; recruitment and allocation methods; follow-up periods; loss to follow-up
(2) Population characteristics: Age, socioeconomic status; HIV disease stage; CD4^+^ cell count; VL; ART status/regimen; comorbidities (e.g., diabetes); obstetric characteristics
(3) Intervention: Mode of delivery; method of determination (e.g., medical records, survey self-report)
(4) Outcomes: Analytic approach; outcome measures and definitions (including both maternal and neonatal outcomes); comparison groups; effect sizes; confidence intervals (CIs); significance levels

Authors were contacted for additional clarification if information in published articles was insufficient.

For randomized controlled trials (RCTs), risk of bias was assessed using the Cochrane Collaboration's tool [9]. This tool assesses random sequence generation (selection bias), allocation concealment (selection bias), blinding of participants and personnel (performance bias), blinding of outcome assessment (detection bias), incomplete outcome data addressed (attrition bias), and selective reporting (reporting bias). Methodological components of the studies were classified as high or low risk of bias. For observational studies using different designs, we adapted the Newcastle–Ottawa scale to consider measures of study quality [10].

### Analysis

We examined results of ECS compared with both vaginal delivery and all other modes of delivery (non-ECS, forceps-assisted or vacuum-assisted delivery, etc.). Although vaginal delivery is the main comparison of interest, in observational studies, this comparison excludes women with medical indications for emergency or non-ECS, potentially biasing results. We therefore also present data for all other modes of delivery.

Where multiple studies reported the same outcome among comparable populations with adequate data, meta-analysis was conducted using random-effects models to combine odds ratios (ORs) using the program Comprehensive Meta-Analysis [11]. Heterogeneity was assessed using the *I*^2^ statistic and interpreted according to Cochrane thresholds [12], and funnel plots were created to examine the potential for publication bias. In meta-analyses, we did not combine data from trials with data from observational studies, as results were expected to differ systematically, resulting in increased heterogeneity [12]. We attempted to identify overlapping participant data across articles by contacting study authors to avoid combining articles with overlapping data in meta-analysis. In cases of overlap, we included only the most recent or comprehensive data in meta-analysis. We conducted stratified analyses for studies conducted in the combination antiretroviral therapy (cART) era (defined as after 1996 or cART use in country). We also conducted stratified analyses of data from women who were on cART and women who had higher CD4^+^ cell counts or lower VLs (defined as CD4^+^ cell count > 200 cells/μl or VL < 400 RNA copies/ml). We then further stratified for women in these categories who delivered their pregnancies at term (at or >37 weeks of gestation) (i.e. cART patients delivering at term, and women with CD4^+^ cell count > 200 cells/μl or VL < 400 RNA copies/ml and delivering at term). Finally, we conducted stratified analyses of data from studies conducted in LMICs, as classified by the World Bank [13].

## Results

### Description of included studies

**Fig. 1:**
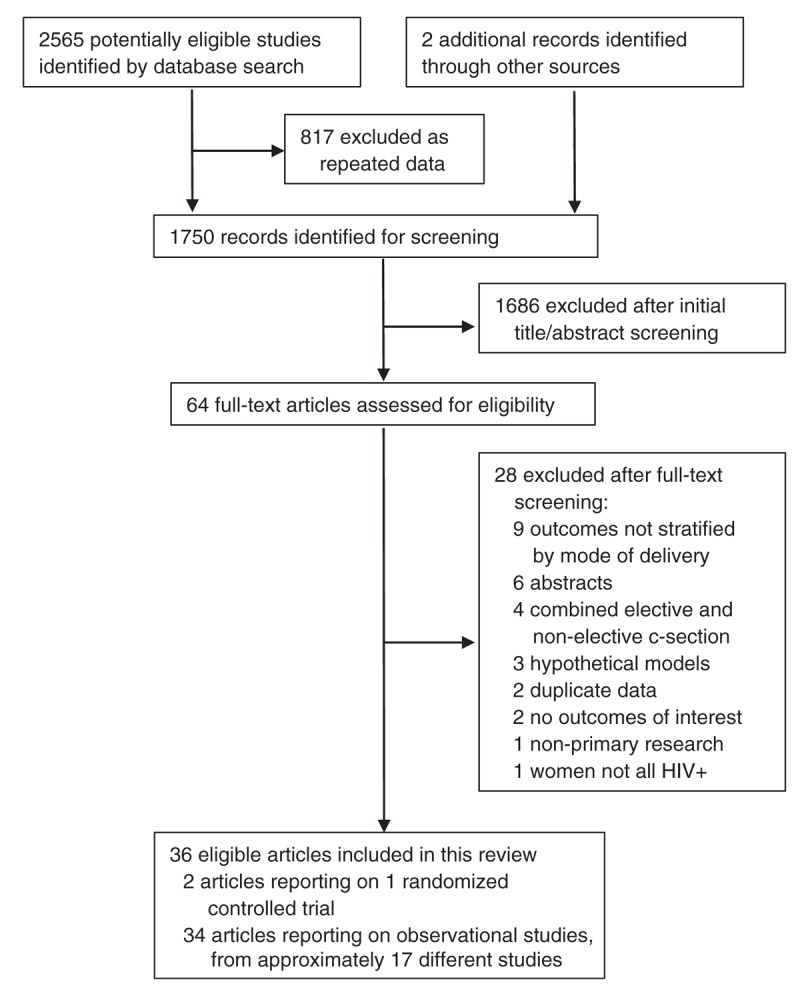
PRISMA flow diagram outlining the article search and selection process.

Figure 1 presents a study selection flowchart. The initial database search yielded 2565 records, with two records identified through other sources; 1750 remained after removing duplicates. After the initial title/abstract review, 64 articles were retained for full-text screening. Ultimately, 36 articles met the inclusion criteria and were included in the review [14–49]. Seventeen were published in 2005 or later (after the cutoff date of the previous review).

Table 1 presents selected characteristics of the 36 included articles. These articles came from approximately 17 different studies; studies overlapped significantly as several long-term cohorts published updated findings, and some contributors to the European Collaborative Study published country-specific cohort subanalyses. Ultimately, data from 25 articles were considered nonoverlapping and are included in the analyses presented below. Settings were mostly in Europe, including data from Belgium, Denmark, France, Germany, Italy, the Netherlands, Poland, Romania, Spain, Sweden, Ukraine, and the United Kingdom. Four studies were conducted in the United States and two in India. One multicountry study (reported in two articles) was conducted in Latin America, whereas individual studies were conducted in Brazil, South Africa, Nigeria, and Kenya.

**Table 1:** Randomized controlled trials and observational studies comparing modes of delivery among women living with HIV.

Study name	First author and year of publication	Study years	Study location (country)	Study design	Sample size (overall)	Follow-up period	Outcome(s)
European Collaborative Study	Bailey 1999 [14]	1987–1998	Belgium, Germany, Italy, Netherlands, Spain, Sweden, United Kingdom	Prospective cohort	364 women, 373 pregnancies	18 months	Infant HIV transmission
European Collaborative Study	European Collaborative Study 2005 [20]	1985–2004	Belgium, Denmark, Germany, Italy, Netherlands, Poland, Spain, Sweden, United Kingdom	Prospective cohort	4525 mother–child pairs	18 months	Infant HIV transmission
European Collaborative Study	European Collaborative Study 2010 [16]	1985–2007	Belgium, Denmark, Germany, Italy, Netherlands, Poland, Spain, Sweden, Ukraine, United Kingdom	Prospective cohort	5238 mother–child pairs	18 months	Infant HIV transmission
European Collaborative Study	Newell 1994 [39]	1986–1992	Belgium, Germany, Italy, Netherlands, Spain, Sweden, United Kingdom	Prospective cohort	1254 mother–child pairs	18 months	Infant HIV transmission
European Collaborative Study	Newell 1996 [40]	1986–1995	Belgium, Germany, Italy, Netherlands, Spain, Sweden, United Kingdom	Prospective cohort	1846 mothers, 1945 children	18 months	Infant HIV transmission
European Collaborative Study	Thorne 2004 [45]	1986–2003	Belgium, Denmark, Germany, Italy, Netherlands, Poland, Spain, Sweden, United Kingdom	Prospective cohort	3231 mother–child pairs	18 months	Infant HIV transmission
European Collaborative Study/European HIV in Obstetrics Group	Fiore 2004 [22]	1992–2002	Italy, Spain, Sweden, Poland, Ukraine	Case-control	408 women	N/A	Maternal morbidities
European Collaborative Study/German Perinatal Cohort	Grosch-Worner 2000 [26]	1985–1999	Germany	Prospective cohort	179 mother–child pairs	18–24 months	Infant HIV transmission
European Collaborative Study/Italy	Grignaffini 2000 [25]	1987–1999	Italy	Prospective cohort	60 women, 64 children	18 months	Infant HIV transmission
European Collaborative Study/Italian Collaborative Study	Italian Collaborative Study 1999 [28]	1988–1990, 1990–1995	Italy	Retrospective cohort (1988–1990), Prospective cohort (1990–1995)	1040 women	18 months	Infant HIV transmission
European Collaborative Study/Italian Register for HIV Infection in Children	Galli 2009 [23]	2002–2004	Italy	Prospective cohort	937 mother–child pairs	18 months	Infant HIV transmission
European Collaborative Study/Italian Register for HIV Infection in Children	Galli 2005 [24]	1985–1995, 1996–2001	Italy	Prospective cohort	4151 children	18 months	Infant HIV transmission
European Collaborative Study/Italian Register for HIV Infection in Children	Italian Register for HIV Infection in Children 2002 [29]	1985–1995, 1996–1999	Italy	Prospective cohort	3770 children	18 months	Infant HIV transmission
European Mode of Delivery Collaboration (EMDC)	EMDC 1999 [21]	1993–1998	Italy, France, Spain	Randomized controlled trial	436 women	18 months	Infant HIV transmission, Maternal morbidities
European Mode of Delivery Collaboration	Ricci 2000 [42]	1993–1998	Italy, France, Spain	Randomized controlled trial	414 women	18 months	Infant HIV transmission, Maternal morbidities
French Perinatal Cohort	Briand 2013 [17]	2000–2010	France	Prospective cohort	8977 women	18 months	Infant HIV transmission, Maternal morbidities
French Perinatal Cohort	Marcollet 2002 [36]	1989–1999	France	Retrospective chart review	401 women	6 weeks	Maternal morbidities
French Perinatal Cohort	Mandelbrot 1998 [35]	1985–1996	France	Prospective cohort	2834 mother–child pairs	18 months	Infant HIV transmission
French Perinatal Cohort	Mandelbrot 2013 [34]	2005–2010	France	Prospective cohort	4654 women	18 months	Infant HIV transmission
IMPAACT Protocol 1025 Study	Livingston 2010 [33]	2002–2008	United States, Puerto Rico	Prospective cohort	1194 mother–child pairs	6 months	Infant HIV transmission, Infant health
Italian Group of the Gynecological and Obstetrics Society	Tibaldi 1994 [46]	1987–1991	Italy	Prospective cohort	519 mothers, 528 children	18 months	Infant HIV transmission
National Study of HIV in Pregnancy and Childhood	Townsend 2014 [47]	2000–2011	UK, Ireland	Population surveillance	12486 mother–child pairs	18 months	Infant HIV transmission
NISDI Perinatal Study	Duarte 2006 [19]	2002–2005	Argentina, Bahamas, Brazil, Mexico	Prospective cohort	697 women	6–12 weeks	Maternal morbidities
NISDI Perinatal/LILAC Studies	Kreitchmann 2011 [32]	2002–2009	Argentina, Bahamas, Brazil, Jamaica, Mexico, Peru	Prospective cohort	1443 mother–child pairs	3 years	Infant health
Pediatric AIDS Clinical Trials Group Protocol 185	Watts 2000 [49]	1993–1997	United States	Prospective cohort	501 women	18 months	Maternal morbidities
Pilot PMTCT Programme	Cocu 2005 [18]	2000–2002	Romania	Prospective cohort	20 women	6 weeks, 3 months, 18 months	Infant HIV transmission
Swiss Neonatal HIV Study Group	Kind 1995 [30]	1986–1993	Switzerland	Prospective cohort	316 children	24 months	Infant HIV transmission
Swiss Neonatal HIV Study Group	Kind 1998 [31]	1986–1996	Switzerland	Prospective cohort	496 children	24 months	Infant HIV transmission
Women and Infants Transmission Study	Navas-Nacher 2006 [38]	1990–2004	United States, Puerto Rico	Prospective cohort	1491 deliveries	2 months, 6 months, 12 months, 18 months	Maternal morbidities
Women and Infants Transmission Study	Read 2001 [41]	1989–1998	United States, Puerto Rico	Prospective cohort	1186 deliveries	2 months	Maternal morbidities
–	Bobat 1996 [15]	1990–1993	South Africa	Prospective cohort	229 women, 234 children	15 months	Infant HIV transmission
–	Iloh 2015 [27]	2011–2012	Nigeria	Prospective cohort	210 children	18 months	Infant HIV transmission
–	Mukherjee 2010 [37]	2001–2005	India	Retrospective cohort	362 women	1 month	Infant HIV transmission
–	Shah 2006 [43]	2000–2003	India	Retrospective cohort	470 mother–child pairs	18 months	Infant HIV transmission
–	Succi 2007 [44]	2000, 2001	Brazil	Cross-sectional (retrospective chart review)	2924 children	15 months	Infant HIV transmission
–	Unger 2014 [48]	2000–2005	Kenya	Prospective cohort	501 women	12 months	Maternal mortality

One study (reported in two articles) was an RCT: the European Mode of Delivery Collaboration [21,42]. The RCT was not blinded (due to the impossibility of blinding mode of delivery), but had limited attrition and received low risk of bias judgments across measures on the Cochrane Risk of Bias tool (see Supplementary Appendix, http://links.lww.com/QAD/B103). The remaining studies were observational designs, mostly prospective cohort studies that followed infants after delivery to assess infant HIV infection outcomes. Follow-up periods ranged from 1 month to 3 years; 27 of the 36 included articles had follow-up periods of 18 months or longer.

Results are presented below for each of the main outcomes. Funnel plots did not indicate publication bias. Heterogeneity was not substantially significant in most meta-analyses.

### Maternal health outcomes

Maternal health outcomes are reported in Table 2. In the RCT, adverse maternal health outcomes were minimal [21,42]. Postpartum fever was reported by 1.1% (2/183) of women who gave birth vaginally and 6.7% (15/225) who gave birth by ECS (*P* = 0.002). Postpartum bleeding or intravascular coagulation disease occurred in one woman in each group. Anemia of greater than moderate severity (hemoglobin < 8 g/dl) was reported in two women who gave birth vaginally and four by ECS. No further adverse events were reported at 6-week follow-up.

**Table 2:** Meta-analytic results for maternal health outcomes, comparing modes of delivery.

	ECS versus vaginal delivery					ECS versus all other modes of delivery				
	No. of articles	No. of effect sizes	No. of participants	OR (95% CI)	*I*^2^	No. of articles	No. of effect sizes	No. of participants	OR (95% CI)	*I*^2^
RCTs										
Postpartum fever	1 [42]	1	414	5.12 (1.48–17.75)^c^	–	–	–	–	–	–
Wound infection	1 [42]	1	414	1.71 (0.31–9.44)^c^	–	–	–	–	–	–
Anesthetic	1 [42]	1	414	4.28 (0.20–89.72)^c^	–	–	–	–	–	–
Anemia	1 [42]	1	414	3.03 (0.62–14.77)^c^	–	–	–	–	–	–
Other complications	1 [42]	1	414	0.63 (0.14–2.85)^c^	–	–	–	–	–	–
Observational studies										
Mortality in the first year postpartum	1 [48]	0^d^	427	–	–	1	0^c^	501	–	–
All morbidities^b^	6 [17,19,22,36,38,49]	7	7821	3.12 (2.21–4.41)	58.14	5 [17,19,36,38,49]	5	7893	1.52 (1.06–2.20)	65.29
All morbidities, LMICs only^a^	1 [19]	1	559	1.16 (0.49–2.71)	N/A^e^	1 [19]	1	697	0.73 (0.35–1.51)	N/A^e^
UTI or febrile UTI	6 [17,19,22,36,41,49]	7	5683	1.85 (1.18–2.88)	0	5 [17,19,36,41,49]	6	6672	1.18 (0.80–1.76)	0
UTI or febrile UTI, LMICs only^a^	1 [19]	1	559	1.57 (0.84–2.92)	N/A^e^	1 [19]	1	697	1.06 (0.62–1.79)	N/A^e^
Endometritis, febrile endometritis, or amnionitis	5 [17,22,36,41,49]	5	5124	1.53 (0.68–3.44)	40.62	4 [17,36,41,49]	4	5975	1.17 (0.65–2.12)	0
Hemorrhage, transfusion, or severe anemia	5 [17,22,36,41,49]	7	5433	1.91 (1.20–3.03)	3.14	4 [17,36,41,49]	6	5975	1.83 (1.07–3.1)	20.76

CI, confidence interval; ECS, elective c-section; LMIC, low-income/middle-income countries; OR, odds ratio; RCT, randomized controlled trial; UTI, urinary tract infection.

^a^Included studies were conducted in countries classified as lower income, lower-middle, or upper-middle income by the World Bank.

^b^All morbidities refers to any major or minor postpartum complication, including abscess, amnionitis, anemia, anesthesia complications, cystitis, deep vein thrombosis, diffuse intravascular dissemination, endometritis, fever, hematoma, hemorrhage, peritonitis, pneumonia, pneumopathy, postpartum operation, pyelonephritis, sepsis, septic pelvic thrombophletitis, septic shock syndrome, subileus, transfusion (red blood cell/platelet), UTI, wound (caesarean incision or episiotomy) infection or dehiscence.

^c^OR and 95% CI were not reported in the text; these statistics were calculated from data presented in tables.

^d^One study presented maternal mortality outcomes. ORs were not calculable given the lack of events in the ECS group. Eight deaths were reported of the 405 women delivering vaginally, five of the 74 given non-ECS, and none of the 22 given ECS.

^e^Not applicable, as meta-analysis was not conducted when there was only a single effect size.

One observational study in Kenya examined maternal mortality [48]. Eight deaths were reported of the 405 women delivering vaginally, five of the 74 given non-ECS, and none of the 22 given ECS [48].

Six observational studies measured overall maternal morbidity (all morbidities combined) [17,19,22,36,38,49]. In meta-analysis, ECS was associated with increased odds of all morbidities compared with vaginal delivery (OR 3.12, 95% CI 2.21–4.41) but the OR was lower when compared with all other modes of delivery (OR 1.52, 95% CI 1.06–2.20) [17,19,36,38,49]. Both meta-analyses demonstrated substantial heterogeneity. Just one of these studies came from LMICs [19]: this multisite study conducted in four Latin American and Caribbean countries found no statistically significant difference in overall maternal morbidity with ECS compared with either vaginal (OR 1.16, 95% CI 0.49–2.71) or all other modes of delivery (OR 0.73, 95% CI 0.35–1.51).

Combining studies measuring UTIs and febrile UTIs, ECS was associated with increased odds of UTIs compared with vaginal delivery (OR 1.85, 95% CI 1.18–2.88) [17,19,22,36,41,49] but not when compared with all other modes (OR 1.18, 95% CI 0.80–1.76) [17,19,36,41,49]. The odds of endometritis, febrile endometritis, and/or amnionitis among women who had ECS was not significantly different from the odds among those with vaginal delivery (OR 1.53, 95% CI 0.68–3.44) [17,22,36,41,49] or all other modes (OR 1.17, 95% CI 0.65–2.12) [17,36,41,49]. Women who had ECS were more likely to have hemorrhage, transfusion, and/or severe anemia compared with women who had vaginal deliveries (OR 1.91, 95% CI 1.2–3.03) [17,22,36,41,49] or all other modes (OR 1.83, 95% CI 1.07–3.1) [17,36,41,49].

### Infant HIV infection

By far, the most common outcome measured was infant HIV infection (Table 3). The RCT found significantly fewer HIV infections among infants delivered by ECS (1.7%) versus vaginal delivery (10.6%) (OR 0.2, 95% CI 0.0–0.5) [42]. The OR was closer to one and nonsignificant for women who received zidovudine in pregnancy (OR 0.4, 95% CI 0–1.4) compared with the OR for women who received no zidovudine in pregnancy (OR 0.2, 95% CI 0–0.8).

**Table 3:** Meta-analytic results for infant HIV infection, comparing modes of delivery across study types and subpopulations.

	ECS versus vaginal delivery					ECS versus all other modes of delivery				
	No. of articles	No. of effect sizes	No. of participants	OR (95% CI)	*I*^2^	No. of articles	No. of effect sizes	No. of participants	OR (95% CI)	*I*^2^
RCTs	1 [42]	1	385	0.2 (0.0–0.5)	N/A^c^	–	–		–	–
Observational studies	13 [15–18,27,31,33,35,37,43,44,46,47]	24	16204	0.43 (0.30–0.63)	40.67	9 [15–17,27,31,33,44,46,47]	19	17638	0.47 (0.33–0.67)	31.26
Studies during the cART era^a^	9 [17,18,20,26,32,35,36,41,45]	19	13719	0.45 (0.30–0.67)	21.89	5 [17,26,32,35,45]	15	15724	0.59 (0.37–0.93)	34.82
cART patients only	4 [17,18,33,47]	13	8823	0.82 (0.47–1.43)	0	3 [17,18,33]	12	12708	0.94 (0.59–1.51)	0
cART patients delivering at term only	1 [17]	5	3269	0.26 (0.62–1.45)	0	1 [17]	5	5242	0.72 (0.35–1.46)	0
Women with CD4^+^ cell count > 200 or VL < 400 only	2 [16,17]	5	4022	0.36 (0.17–0.79)	10.08	2 [16,17]	5	6314	0.46 (0.24–0.88)	0
Women with CD4^+^ cell count > 200 or VL < 400 delivering at term only	1 [17]	2	2782	0.59 (0.21–1.63)	0	1 [17]	2	4644	0.73 (0.29–1.80)	0
LMICs only^b^	5 [18,27,37,43,44]	6	2925	0.27 (0.16–0.45)	18.56	2 [27,44]	3	3016	0.34 (0.15–0.78)	67.32

cART, combination antiretroviral therapy; CI, confidence interval; ECS, elective c-section; LMIC, low-income/middle-income countries; OR, odds ratio; RCT, randomized controlled trial; VL, viral load.

^a^cART era was considered after 1996 or ART use in country.

^b^Countries classified as lower income, lower-middle, or upper-middle income by the World Bank.

^c^Not applicable, as meta-analysis was not conducted on the single RCT.

In meta-analysis of all observational studies, ECS was also associated with a decreased odds of infant HIV infection (Table 3). The OR for infant HIV infection comparing ECS to vaginal delivery was 0.43 (95% CI 0.30–0.63, moderate heterogeneity) [15–18,27,31,33,35,37,43,44,46,47] and 0.47 (95% CI 0.33–0.67, moderate heterogeneity) when comparing to all other modes of delivery [15–17,27,31,33,44,46,47]. For studies conducted during the cART era, ECS continued its association with decreased odds of infant HIV infection.

Stratifying to patients receiving cART, the relationship between ECS and lower infant HIV infection was no longer statistically significant (OR 0.82, 95% CI 0.47–1.43 versus vaginal delivery [17,18,33,47]; OR 0.94, 95% CI 0.59–1.51 versus all other modes [17,18,33]). Examining data from cART patients delivering at term also yielded nonsignificant results (Table 3) [17].

When focusing on data stratified by CD4^+^ or VL of the mother, only two studies [16,17] (with five individual effect sizes) were available. Among women with CD4^+^ cell count more than 200 or VL less than 400 only, the OR for infant HIV infection was 0.36 (95% CI 0.17–0.79) compared with vaginal delivery [16,17] and 0.46 (95% CI 0.24–0.88) compared with all other modes [16,17]. However, in both comparisons, there was no longer a statistically significant association between ECS and infant HIV infection when examining data for women with CD4^+^ cell count more than 200, VL less than 400, and delivery at term only (OR 0.59, 95% CI 0.21–1.63 versus vaginal delivery; OR 0.73, 95% CI 0.29–1.8 versus all other modes of delivery) [17].

Finally, meta-analysis of data from LMICs showed that ECS was associated with reduced infant HIV infection compared with vaginal delivery (OR 0.27, 95% CI 0.16–0.45) and all other modes of delivery (OR 0.34, 95% CI 0.15–0.78, substantial heterogeneity) [18,27,37,43,44].

### Other infant health outcomes

Two observational studies from the United States and Puerto Rico [33] and from multiple countries in Latin America and the Caribbean [32] compared infant health outcomes in addition to HIV infection (Table 4). Odds of infant respiratory distress syndrome increased with ECS compared with vaginal delivery but not with all other modes of delivery. ECS had no statistically significant difference in odds of transient tachypnea comparing with vaginal delivery (substantial heterogeneity) but increased odds comparing with all other modes. Results from the single study conducted in LMICs (multiple countries in Latin America and the Caribbean) were similar to meta-analytic results for infant respiratory distress syndrome, but showed greater odds of transient tachypnea compared with vaginal delivery (OR 7.10, 95% CI 2.09–24.12) [32]. No studies compared breastfeeding outcomes across modes of delivery.

**Table 4:** Meta-analytic results for other infant health outcomes, comparing modes of delivery.

	ECS versus vaginal delivery				ECS versus all other modes of delivery			
	No. of studies	No. of participants	OR (95% CI)	*I*^2^	No. of studies	No. of participants	OR (95% CI)	*I*^2^
Observational studies								
Respiratory distress syndrome	2 [32, 33]	2056	2.77 (1.58–4.88)	0	2 [32, 33]	2637	1.43 (0.94–2.18)	0
Respiratory distress syndrome, LMICs only^a^	1 [32]	1078	2.73 (1.24–5.98)	–	1 [32]	1443	1.48 (0.83–2.63)	–
Transient tachypnea of the newborn	2 [32, 33]	2056	3.17 (0.79–12.76)	74.39	2 [32, 33]	2637	1.73 (1.09–2.74)	0
Transient tachypnea of the newborn, LMICs only^a^	1 [32]	1078	7.10 (2.09–24.12)	–	1 [32]	1443	1.73 (0.91–3.27)	–

OR, odds ratio; CI, confidence interval; ECS, elective c-section; LMIC, low-income/middle-income countries.

^a^Countries classified as lower income, lower-middle, or upper-middle income by the World Bank.

## Discussion

This systematic review identified a large body of evidence comparing outcomes across different modes of delivery for women living with HIV. However, most studies were conducted in high-income countries and among women who were not on current highly effective ART regimens. Altogether, data from a single RCT and multiple observational studies indicate that ECS reduces the risk of infant HIV infection in the absence of ART. However, the association between ECS and infant HIV infection was nonsignificant in most stratified analyses of studies conducted in the cART era and among women on cART, women with higher CD4^+^ cell counts or lower VLs, and women whose deliveries were at term. Limited data on other maternal outcomes and infant health outcomes do suggest increased maternal and infant morbidity associated with ECS compared with vaginal birth, as is seen with HIV-uninfected women. However, many outcomes were relatively minor or less problematic with accurate dating of pregnancy and ECS at term.

The risk–benefit ratio of ECS likely depends upon the underlying rate of vertical HIV transmission, as well as the risks of both maternal and infant morbidities and mortality associated with ECS and other modes of delivery. For women who are on ART and virally suppressed, the risk of vertical HIV transmission is relatively low. For women in high-income countries with access to quality obstetric services, the risks associated with ECS are also relatively low. However, the risk of vertical transmission increases greatly for women in the absence of effective ART while the risks of ECS increase for women without access to high-quality obstetric services. We found only three studies from sub-Saharan Africa, and whereas one study from Kenya reported on maternal mortality, none reported on maternal or infant morbidity outcomes other than HIV infection. Future studies from sub-Saharan Africa and other LMICs would help to clarify the risks and benefits in such settings and provide useful evidence for policy-makers.

The findings from this review suggest routine ECS for women living with HIV may not be appropriate; instead, individual patients and clinicians should consider the risks and benefits for specific clients, and women's autonomy to choose their mode of delivery should be respected. This is consistent with other national guidelines and recommendations from professional groups [50–53]. US and UK guidelines, while not recommending routine ECS for all women living with HIV, do recommend that clinicians consider ECS at higher VLs. The American College of Obstetricians and Gynecologists has recommended considering ECS when VL more than 1000 [51]. UK guidelines recommend ECS with VL more than 1000 and recommend considering ECS when VL = 50–999, ‘taking into account the actual VL, the trajectory of the VL, length of time on treatment, adherence issues, obstetric factors and the woman's views’ [53]. An examination of national guidelines across 23 European countries found that 95% ‘included the recommendation that HIV-positive women on successful cART with a very low or undetectable VL (<1000) can have a vaginal delivery’ [50]. The 2015 WHO Statement on Cesarean Section Rates emphasized the need to avoid unnecessary c-sections, especially in settings that lack the facilities and/or capacity to properly conduct safe surgery and treat surgical complications, which can extend many years beyond the current delivery and affect the health of the woman, her child, and future pregnancies [3]. However, there may be specific clinical indications, such as raised VL at delivery or known ART resistance, where ECS may be further considered, highlighting the need for individual-level consideration of risks and benefits of ECS in addition to national guidelines. When c-section is medically indicated, it should be available, accessible, and safe for all women, including women living with HIV.

It is critically important to emphasize respect for women's autonomy regarding mode of delivery. In the largest survey conducted by and for women living with HIV globally, women living with HIV reported experiencing routine lack of inclusion or choice in decision-making about their own sexual and reproductive healthcare [6]. Principles of human rights must be embedded in all healthcare policies, practices, and training, and coercion of any kind is never acceptable [54].

The issue of mode of delivery for women living with HIV is important, and no systematic review has been conducted to update the evidence in the past 12 years. Our review used a broad search strategy, double data extraction, and careful assessment of study quality. However, the findings must be seen in light of several limitations. Studies that defined ECS used a definition consistent with the one used for this review; however, the minority of studies that did not clearly specify how they defined ECS may have introduced heterogeneity into the review. Few studies were available from recent years, from women on cART, and for different subgroups. Few studies were also available from LMICs where surgical skills and health system capacity are most limited; we identified only three studies from sub-Saharan Africa and none reported maternal and infant morbidity outcomes beyond HIV transmission. All but one were observational studies with their well established and inherent limitations and bias in assessing intervention effects; in the absence of randomization, providers likely directed women to ECS or other modes of delivery based on systematically different sociodemographic characteristics, clinical presentation, or staffing capabilities. The only RCT was published in 1999. In meta-analyses, we attempted to include only nonoverlapping participant data, but the complex set of overlaps across studies made this difficult, and it is possible that duplicate data were included in some analyses. Meta-analyses also often had few studies, large CIs, and sometimes considerable statistical heterogeneity. This review points to the need for further research, particularly in low-income and middle-income countries, and particularly in sub-Saharan Africa where the majority of women living with HIV reside. However, reductions in vertical transmission due to cART mean future studies must be large (and thus expensive) to identify statistically significant differences across modes of delivery. The evidence base is therefore unlikely to be significantly strengthened in the future.

In conclusion, our findings suggest that while ECS may be protective against infant HIV infection in the absence of effective ART, this effect was not statistically significant among women on cART or who are at term and virally suppressed, and there are other risks to mothers and infants associated with ECS. Risks and benefits are likely to differ across settings. Clinicians and healthcare providers should consider the risks and benefits for individual clients, and respect women's autonomy to choose their mode of delivery.

## Acknowledgements

This review was commissioned by the WHO, Department of Reproductive Health and Research, to inform the updated WHO consolidated guideline on sexual and reproductive health and rights of women living with HIV. We would like to thank Marie-Louise Newell for her insight on the European Collaborative Study overlapping cohorts and Jennifer Read for her careful review and feedback on the original review protocol and final manuscript.

C.E.K., A.P.B., and M.N. conceived the study and developed the review methods and protocol. C.E.K. performed the literature search and oversaw screening and data extraction. P.T.Y. and S.P. extracted data. C.E.K., P.T.Y., and S.P. conducted the analysis. C.E.K. led the writing of the manuscript with significant help from P.T.Y. S.P., A.P.B., and M.N. commented on and contributed to the text. All authors reviewed and approved the final manuscript.

The study was funded by the WHO.

### Conflicts of interest

The views and opinions expressed herein are those of the authors and not necessarily those of the WHO.

The authors declare no competing interests.
